# Retinoblastoma cell-derived exosomes promote angiogenesis of human vesicle endothelial cells through microRNA‐92a-3p

**DOI:** 10.1038/s41419-021-03986-0

**Published:** 2021-07-13

**Authors:** Shuilian Chen, Xi Chen, Qian Luo, Xuan Liu, Xiao Wang, Zedu Cui, Anqi He, Shengyu He, Zihua Jiang, Nandan Wu, Pei Chen, Keming Yu, Jing Zhuang

**Affiliations:** State Key Laboratory of Ophthalmology, Zhongshan Ophthalmic Center, Sun Yat-sen University, No.7 Jinsui Road, Tianhe District, Guangzhou City, China

**Keywords:** Paediatric cancer, Tumour angiogenesis, Tumour biomarkers

## Abstract

Exosomes derived from tumor cells play a key role in tumor development. In the present study, we identified the bioactivity of exosomes released from WERI-Rb1 retinoblastoma cells in tumor angiogenesis, as well as the underlying mechanism, through biochemical methods and animal experiments. Our in vitro data showed that exosomes could be engulfed by human vesicle endothelial cells (HUVECs), significantly promote cell viability and induce an inflammatory response in HUVECs by increasing the expression of a series of related genes, such as IL-1, IL-6, IL-8, MCP-1, VCAM1, and ICAM1. Significant increases in migration and tube formation were also observed in the HUVECs incubated with exosomes. Moreover, experiments with a nude mouse xenotransplantation model showed that exosomes injected near tumors could be strongly absorbed by tumor cells. The numbers of endothelial cells and blood vessels were significantly increased in tumor tissues treated with exosomes compared to control tissues. Furthermore, to reveal the mechanism underlying exosome-mediated angiogenesis in retinoblastoma, we analyzed the levels of 12 microRNAs in the exosomes. Specifically, our data showed that miR-92a-3p was enriched in RB exosomes. Accordingly, miR-92a-3p was increased in the HUVECs incubated with these exosomes. After treatment with a miR-92a-3p inhibitor, the promoting effect of exosomes on the migration and tube formation of HUVECs was significantly abrogated. The expression of the angiogenesis-related genes mentioned above was markedly decreased in HUVECs. Similarly, treatment with a microRNA mimic also demonstrated that miR-92a-3p was involved in the angiogenesis of HUVECs. More importantly, bioinformatics analysis predicted that Krüppel-like factor 2 (KLF2), a member of the KLF family of zinc-finger transcription factors, might be an active target of miR-92a-3p. Notably, this prediction was confirmed both in vitro and in vivo. Thus, our work suggests that exosomal miR-92a-3p is involved in tumor angiogenesis and might be a promising therapeutic candidate for retinoblastoma.

## Introduction

Retinoblastoma (RB), a primary pediatric intraocular cancer, threatens the health of children, and its clinical management is still challenging [1]. Forty percent of RB cases are caused by the loss or mutation of both alleles of the RB gene (Rb1) during retinal development. Sixty percent of patients have the nonhereditary form of RB [2]. The current treatments for RB include enucleation, radiation therapy, and systemic chemotherapy. However, the prognosis of patients who receive these therapies remains poor in developing countries and depends on the stages of RB [1, 3]. In addition, angiogenesis, which provides a means of tumor metastasis through the blood and promotes tumor invasion, is one of the most important indicators of the biological behavior of malignancies [4]. Previous studies have reported that angiogenesis is crucial for RB progression is a factor that determines disease dissemination [5, 6]. However, angiogenesis is a complicated, multistep process involving extracellular matrix remodeling, endothelial progenitor cell migration and proliferation, capillary differentiation, and anastomosis [7]. The mechanism by which angiogenesis is activated in RB tumor cells is not yet well defined.

Exosomes are small membrane-bound vesicles secreted by various types of cells that have a diameter of 40–100 nm and contain multiple biological molecules, such as proteins, lipids, mRNAs, and microRNAs; exosomes play an important role in intercellular communication and cell signal transduction [8, 9]. Exosomes can deliver genetic information to the target cells by fusion with the plasma membrane, and accumulating evidence has proven that exosomes released by tumor cells participate in different pathological stages of angiogenesis [10], immunosuppression [11], and tumor deterioration [12]. Our previous study demonstrated that exosomes derived from WERI-Rb1 cells could enhance tumor progression by infiltrating the microenvironment [13]. However, the mechanism underlying exosome-mediated tumor progression is not clear. In addition, increasing evidence has demonstrated that microRNAs in exosomes contribute to these effects. Moreover, different microRNAs play a key role in the pathology of tumor tissues. For example, miR-210 in hepatocellular carcinoma (HCC)-derived exosomes can directly inhibit the expression of SMAD4 and STAT6, promoting angiogenesis [14]. In addition, the miRNA-25-3p from colorectal cancer (CRC) dramatically facilitates vascular leakiness and enhances CRC metastasis [15]. Whether microRNAs in exosomes derived from RB affect RB angiogenesis has not been reported.

To address the questions above, we investigated the potential function of RB-derived exosomes both in vitro and in vivo. First, the bioactivity of exosomes on HUVECs was detected by assaying angiogenic factor expression, migration, and tube formation in vitro. The results were confirmed in a murine xenograft model of RB. Second, our study demonstrated that RB exosome-derived miR-92a-3p plays a crucial role in angiogenesis. Moreover, the target gene of miR-92a-3p was predicted by bioinformatics analysis and confirmed by biochemical methods. Therefore, this study focused on the identification of the agents that target tumor angiogenesis to affect RB progression and prognosis.

## Results

### Exosomes derived from WERI-Rb1 cells promote angiogenesis by increasing the inflammatory environment in vitro

**Fig. 1:**
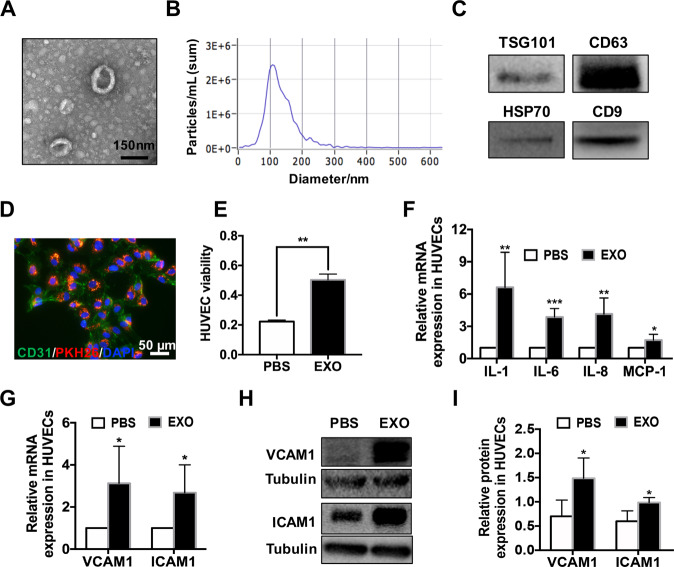
Exosomes derived from WERI-Rb1 cells mediate the activity of HUVECs. **A** Representative TEM images of WERI-Rb1 exosomes. The diameter of the vesicles was approximately 50–150 nm. Bar = 150 nm. **B** Nanoparticle tracking analysis (NTA) indicated that the average size and number of exosomes (97.7% of exosomes had a size of 108.0 nm). **C** Western blot analysis of the different protein markers (HSP70, CD9, TSG101, CD63) of exosomes collected from the WERI-Rb1 cells. **D** Exosomes labeled with PKH26 were swallowed by HUVECs (PKH26, red; CD31, green; DAPI, blue). Bar = 50 μm. **E** CCK-8 assays showed that the viability of the HUVECs was markedly elevated by the exosomes (*n* = 3, **P < 0.01). **F** Real-time PCR analysis of IL-1, IL-6, IL-8 and MCP-1 expression in the HUVECs incubated with the exosomes derived from WERI-Rb1 cells for 24 h. (*n* = 6, *P < 0.05, **P < 0.01, ***P < 0.001) according to two-tailed Student’s *t* test. **G** Real-time PCR analysis of VCAM1 and ICAM1 expression in the HUVECs incubated with the exosomes derived from WERI-Rb1 cells for 24 h (*n* = 6, *P < 0.05). **H** Western blot analysis indicating that exosome treatment upregulated VCAM1 and ICAM1 expression in the HUVECs. **I** Relative quantification of protein expression in HUVECs was performed by densitometry (*n* = 4, *P < 0.05).

Transmission electron microscopy showed that exosomes isolated from WERI-Rb1 cells, as described in the “Materials and methods” section, were round vesicles with bilayer membranes and a diameter of 50–150 nm (Fig. 1A). As shown in Fig. 1B, nanoparticle tracking analysis (NTA) also indicated the average size and number of exosomes (97.7% of exosomes had a size of 108.0 nm). In addition, TSG101, CD63, HSP70, and CD9 were markers used to identify the exosomes, and the expression of these molecules was confirmed by western blotting (Fig. 1C).

Given the importance of angiogenesis in the metastasis of tumors, human umbilical vein endothelial cells (HUVECs) were incubated with exosomes derived from WERI-Rb1 cells. At 6 h after incubation, the cells treated with exosomes labeled with PKH26 (red) were stained with CD31, a marker of endothelial cells. As shown in Fig. 1D, the exosomes (red) were mainly located in the cytoplasm. Moreover, at 24 h after incubation, compared with PBS, the exosomes significantly increased the viability of HUVECs (PBS, 0.223 ± 0.015; EXO, 0.570 ± 0.100; **P < 0.01) (Fig. 1E).

Furthermore, we evaluated the expression levels of inflammatory cytokines involved in the angiogenic process, such as IL-1, IL-6, IL-8, and MCP-1 [16]. As shown in Fig. 1F, compared to PBS, the exosomes significantly increased the mRNA levels of IL-1, IL-6, IL-8, and MCP-1 in the HUVECs (relative fold change: IL-1, 6.62 ± 3.27-fold; IL-6, 3.86 ± 0.79-fold; IL-8, 4.14 ± 1.50-fold; MCP-1, 1.73 ± 0.53-fold; *P < 0.05, **P < 0.01, ***P < 0.001). Accordingly, the mRNA levels of VCAM1 and ICAM1, which are adhesion molecules that are emerging as important genes in angiogenesis, were also significantly increased (VCAM1, 3.13 ± 1.76-fold, *P < 0.05; ICAM1, 2.68 ± 1.32-fold, *P < 0.05) (Fig. 1G).

To confirm the protein expression of VCAM1 and ICAM1, total protein was extracted from HUVECs at 24 h after treatment with the exosomes or PBS. VCAM1 and ICAM1 expression was significantly higher in the cells incubated with the exosomes than in the control cells (VCAM1: PBS, 0.701 ± 0.335; EXO, 1.486 ± 0.420, *P < 0.05. ICAM1: PBS, 0.599 ± 0.215; EXO, 0.987 ± 0.102, *P < 0.05) (Fig. 1H, I). These data are consistent with the mRNA expression results (Fig. 1G).

**Fig. 2:**
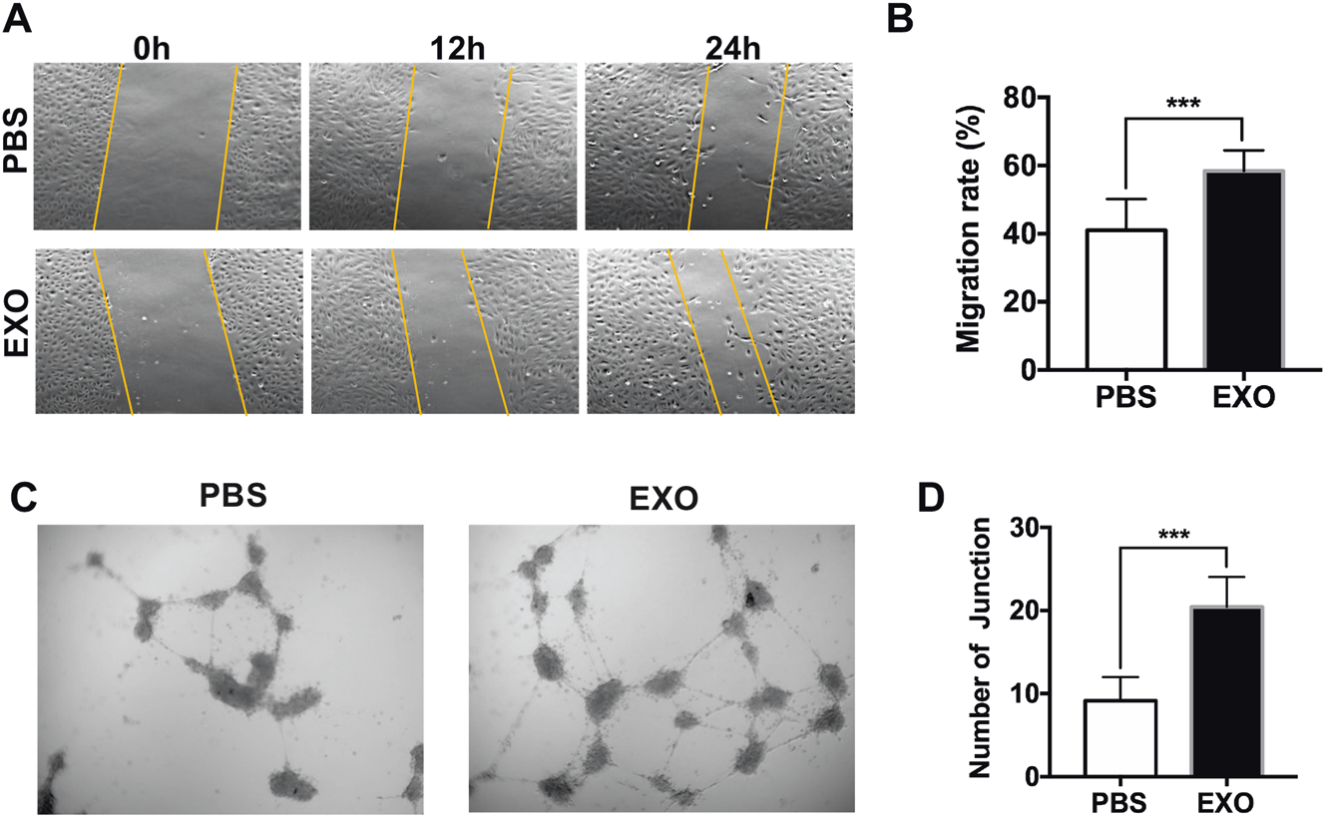
Exosomes derived from WERI-Rb1 cells induce HUVEC angiogenesis and migration in vitro. **A** Wound-healing assay. HUVECs were treated with exosomes, and cell monolayers were scratched with 1–200-μl yellow tips. Images were captured at 0, 12, and 24 h after the wound scratch. **B** The rate of migration was measured by quantifying the total distance that the HUVECs moved from the edge of the scratch toward the center of the scratch. Relative quantification of HUVEC migration 24 h after the wound scratch (*n* = 7, ***P < 0.001). **C** Microscopic photographs of tubule formation on Matrigel are shown. There was a significant increase in HUVEC tubule connections in the presence of the exosomes compared with that in the presence of PBS. **D** The number of tubule junctions is presented in the histograms (*n* = 6, ***P < 0.001).

The expression levels of the genes mentioned above suggest that exosomes promote HUVEC migration. Consistent with this hypothesis, as shown in Fig. 2A, a scratch wound assay indicated that a smaller wound area remained, as observed by microscopy, at 12 and 24 h after treatment with exosomes than after treatment with PBS. Figure 2B shows the quantification of the HUVEC migration rate at 24 h (PBS, 41.04 ± 0.09%; EXO, 58.43 ± 0.060%, **P < 0.01). Accordingly, compared with PBS treatment, exosome treatment significantly increased capillary-like tubes and junctions (PBS, 9.375 ± 2.722; EXO, 22.000 ± 5.244; ***P < 0.001) (Fig. 2C, D). Taken together, these results show that exosomes can induce an inflammatory response in HUVECs, suggesting a possible link between exosomes and angiogenesis.

### Exosomes derived from WERI-Rb1 cells increase retinoblastoma growth and angiogenesis in vivo

**Fig. 3:**
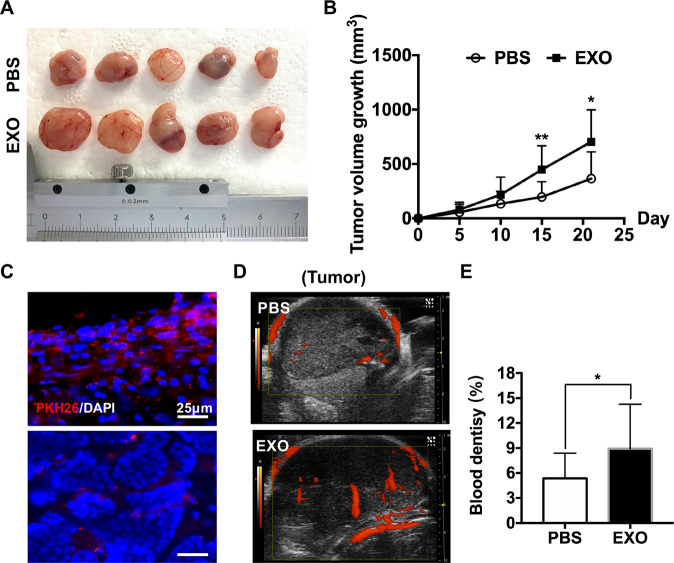
Exosomes derived from WERI-Rb1 cells were engulfed by tumor cells and promoted the growth of retinoblastoma. **A** Representative macroscopic appearance of tumors after subcutaneously injection PBS or exosomes for 21 days. **B** The tumor volume growth curve in the xenotransplantation model (*n* = 14, ^*^P < 0.05, ^**^P < 0.01). **C** PKH26-labeled exosomes (red) were internalized by cells from tumor tissues after 10 days after exosomes injection. DAPI was used to stain the nuclei. Bar = 25 μm. **D** Ultrasound analysis showed that blood flow was increased by exosome injection. **E** Quantification of blood flow in tumors (*n* = 9, ^*^P < 0.05).

To confirm the results in vitro, we investigated whether exosomes from WERI-Rb1 cells promote angiogenesis in vivo. We subcutaneously injected WERI-Rb1 cells into Balb/c mice. The mice successfully implanted with tumors were randomly divided into the PBS or exosome groups one week after tumor cell injection. The tumor volumes were calculated every 5 days with the following formula: length × width^2^/2. Mice were sacrificed and tumors were collected after 21 days of PBS or exosomes injection. Five representative tumors in each group were shown in Fig. 3A. The tumor volume growth in the exosome group was significantly larger than that of PBS group at day 15 and day 21 (tumor volume growth: D15, PBS, 197.26 ± 140.82 mm^3^, EXO, 448.47 ± 220.36 mm^3^; D21, PBS, 365.77 ± 247.00 mm^3^, EXO, 704.47 ± 294.09 mm^3^, *P < 0.05, **P < 0.01) (Fig. 3B).

After 10 days of exosome injection, the PKH26-labeled exosomes were observed in the margins of the tumors and dense tissue within the tumors, indicating that exosomes could be absorbed by the tumors (Fig. 3C). We also detected the effect of the exosomes on angiogenesis in vivo using ultrasound imaging. As shown in Fig. 3D, the exosomes substantially increased the blood flow in the xenografted tumors, which is consistent with the in vitro data. According to the blood flow ratio in each ultrasound image analyzed by the software, there was a significant increase in the exosome-injected groups compared to the control group (PBS, 10.00 ± 5.69%; EXO, 5.61 ± 3.35%, *P < 0.05) (Fig. 3E).

**Fig. 4:**
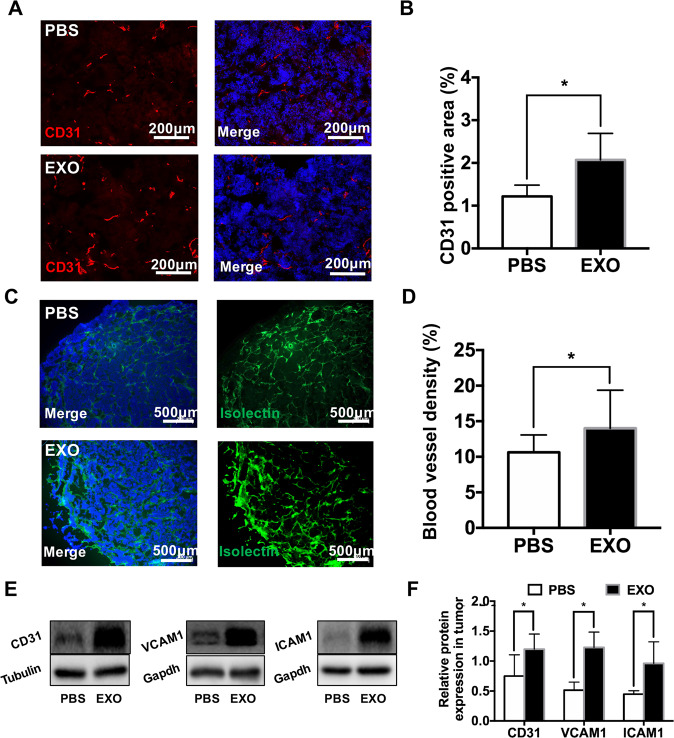
Exosomes derived from WERI-Rb1 cells increase retinoblastoma angiogenesis in vivo. **A** Immunofluorescence analysis of CD31-positive cells showed that the capillary density in the EXO-injected group was increased. Bar = 200 μm. **B** Quantification of the CD31-positive area (%) in the tumors (*n* = 5, ^*^P < 0.05). **C** Immunofluorescence analysis of the isolectin positive areas. Bar = 500 μm. **D** Quantification of isolectin positive area (blood vessel density, %) in the tumors (*n* = 7, ^*^P < 0.05). **E** Western blot images showing the protein levels of CD31, VCAM1, and ICAM1 in tumors from the PBS-injected group and EXO-injected group. **F** Relative quantification of protein expression in tumors (*n* = 6, *P < 0.05).

Moreover, the tumor sections were stained with antibodies against CD31, which binds to endothelial cells. More positive cells were observed in tissues injected with exosomes (PBS, 1.21 ± 0.26%; EXO, 2.07 ± 0.62%, *P < 0.05;) (Fig. 4A, B). In addition, we used the isolectin B4 to visualize blood vessels in the tumor tissues [17] (Fig. 4C). The statistical data showed that the exosomes significantly increased the isolectin positive areas (PBS, 10.64 ± 1.41%; EXO, 15.46 ± 3.23%, *P < 0.05) (Fig. 4D). Furthermore, Western blot analysis revealed that the protein levels of CD31, VCAM1 and ICAM1 were significantly increased by the exosomes (CD31: PBS, 0.749 ± 0.357, EXO, 1.200 ± 0.251; VCAM1: PBS, 0.515 ± 0.134, EXO, 1.228 ± 0.256; ICAM1, PBS: 0.447 ± 0.058, EXO: 0.961 ± 0.360, *P < 0.05) (Fig. 4F).

Taken together, these findings demonstrate that exosomes derived from WERI-Rb1 cells can significantly increase RB growth and angiogenesis.

### Exosomally delivered miR-92a-3p mediates proangiogenic activity in vitro

**Fig. 5:**
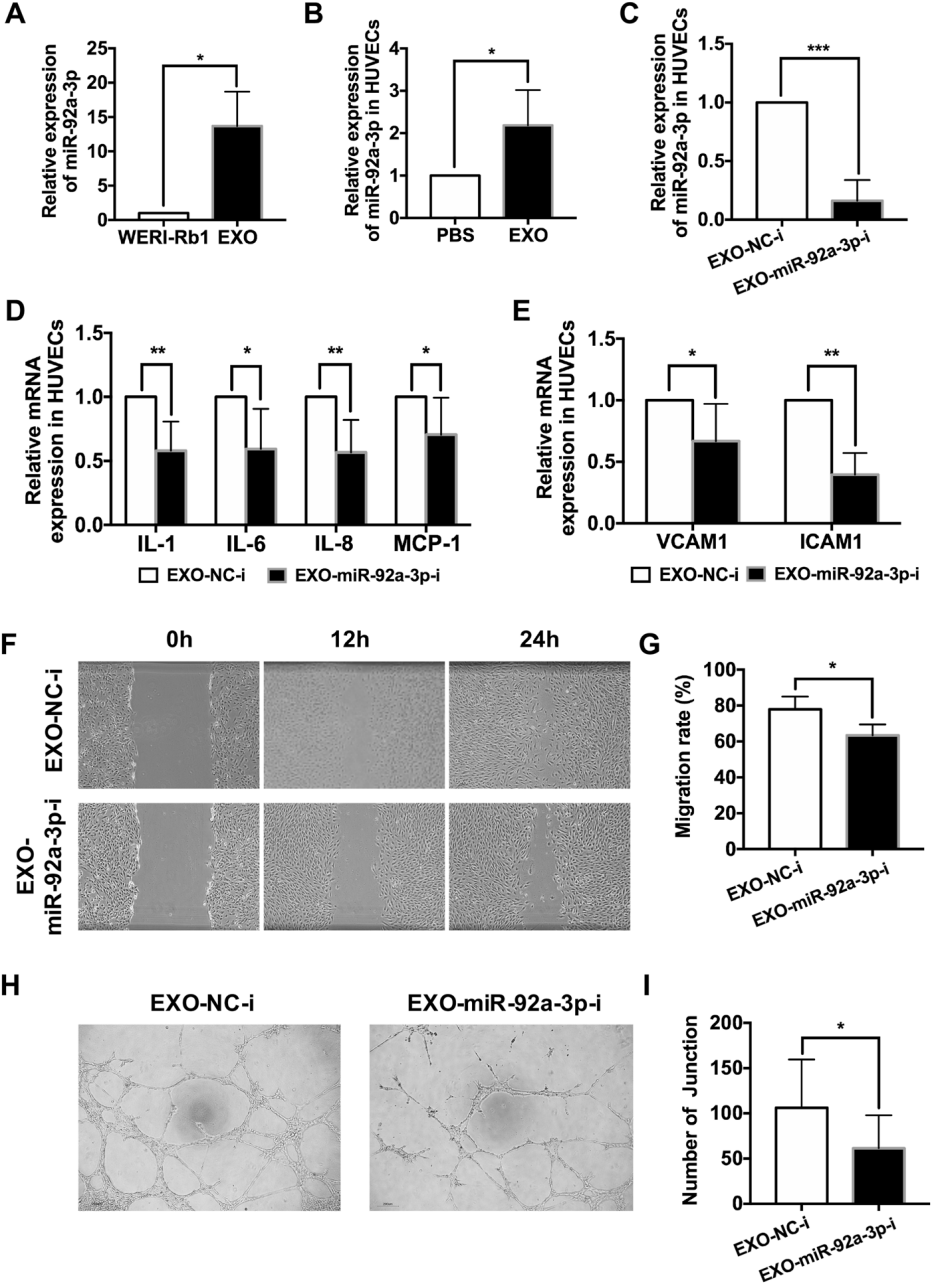
Exosomal miR-92a-3p promotes HUVEC angiogenesis and migration in vitro. **A** miR-92a-3p showed higher expression in the EXOs than in the WERI-Rb1 cells (*n* = 3, ^*^P < 0.05). **B** miR-92a-3p was increased in the HUVECs treated with EXOs (*n* = 5, ^***^P < 0.001). **C–E** HUVECs were incubated with EXOs transfected with synthetic inhibitor control (EXO-NC-i) or miR-92a-3p inhibitor (EXO-miR-92a-i). The mRNA levels of miR-92a-3p, VCAM1, ICAM1, IL-1, IL-6, IL-8, and MCP-1 were decreased (*n* = 7, ^*^P < 0.05, ^**^P < 0.01). **F** Wound-healing assay revealed that EXO-miR-92a-i could inhibit the migration rate of the HUVECs. **G** Relative quantification of HUVEC migration 24 h after the wound scratch (*n* = 4, ^*^P < 0.05). **H**, **I** Quantitative evaluation of number of the junctions after treating the HUVECs with EXO-NC-i or EXO-miR-92a-i (24 h, *n* = 10, ^*^P < 0.05).

To identify potential angiogenesis-related miRNAs in RB exosomes, we analyzed the expression of some miRNAs, such as miR-92a-3p, miR-16, miR-17a-3p, miR-17a-5p, miR-18a-5p, miR-18a-3p, miR-34a-5p, miR-34a-3p, miR-93a-5p, miR-93a-3p, miR-20a-5p, and miR-129-5p, in RB cells and exosomes derived from RB cells using real-time RT-PCR. These miRNAs were previously reported to be factors involved in angiogenesis in other tumors [18, 19]. Fortunately, we found that miR-92a-3p was significantly higher in the exosomes than in the RB cells (relative fold change: 13.68 ± 5.01-fold, *P < 0.05) (Fig. 5A), which indicates that miR-92a-3p could be enriched by exosomes. The expression of other miRNAs was not significantly different between the RB cells and the exosomes derived from RB cells (data was not shown). Moreover, the exosomes from RB cells increased the relative expression of miR-92a-3p in HUVECs (relative fold change: 2.19 ± 0.83-fold, *P < 0.05) (Fig. 5B).

To further evaluate the effect of miR-92a-3p in exosomes on angiogenesis, we knocked down miR-92a-3p in exosomes using a miR-92a-3p inhibitor (miR-92a-3p-i) (relative fold change: 0.16 ± 0.18-fold, ***P < 0.001) (Fig. 5C). Consistent with our hypothesis, the exosomes with miR-92a-3p knocked down significantly decreased the inflammatory cytokine mRNA levels in HUVECs compared to the control exosomes (relative fold change: IL-1, 0.58 ± 0.23-fold; IL-6, 0.59 ± 0.31-fold; IL-8, 0.57 ± 0.25-fold; MCP-1, 0.71 ± 0.29-fold; *P < 0.05, **P < 0.01) (Fig. 5D). Accordingly, a significant decrease in proangiogenic factors was observed in the HUVECs incubated with the exosomes with miR-92a-3p knocked down (VCAM1, 0.67 ± 0.30-fold; ICAM1, 0.40 ± 0.18-fold) (Fig. 5E). Wound-healing and tube-formation assays also showed that miR-92a-3p plays a critical role in exosome-mediated angiogenesis. As shown in Fig. 5F, G, the migration rate of the HUVECs treated with exosomes containing a miR-92a-3p inhibitor was significantly diminished at 24 h (the migration rate: EXO-NC-i, 77.97 ± 7.04%; EXO-miR-92a-3p-i, 63.34 ± 6.13%, *P < 0.05). The number of junctions was also significantly decreased too (EXO-NC-i, 106.30 ± 53.23; EXO-miR-92a-3p-i, 61.35 ± 36.51, *P < 0.05) (Fig. 5H, I).

### Overexpression of miR-92a-3p in HUVECs enhances angiogenesis in vitro

**Fig. 6:**
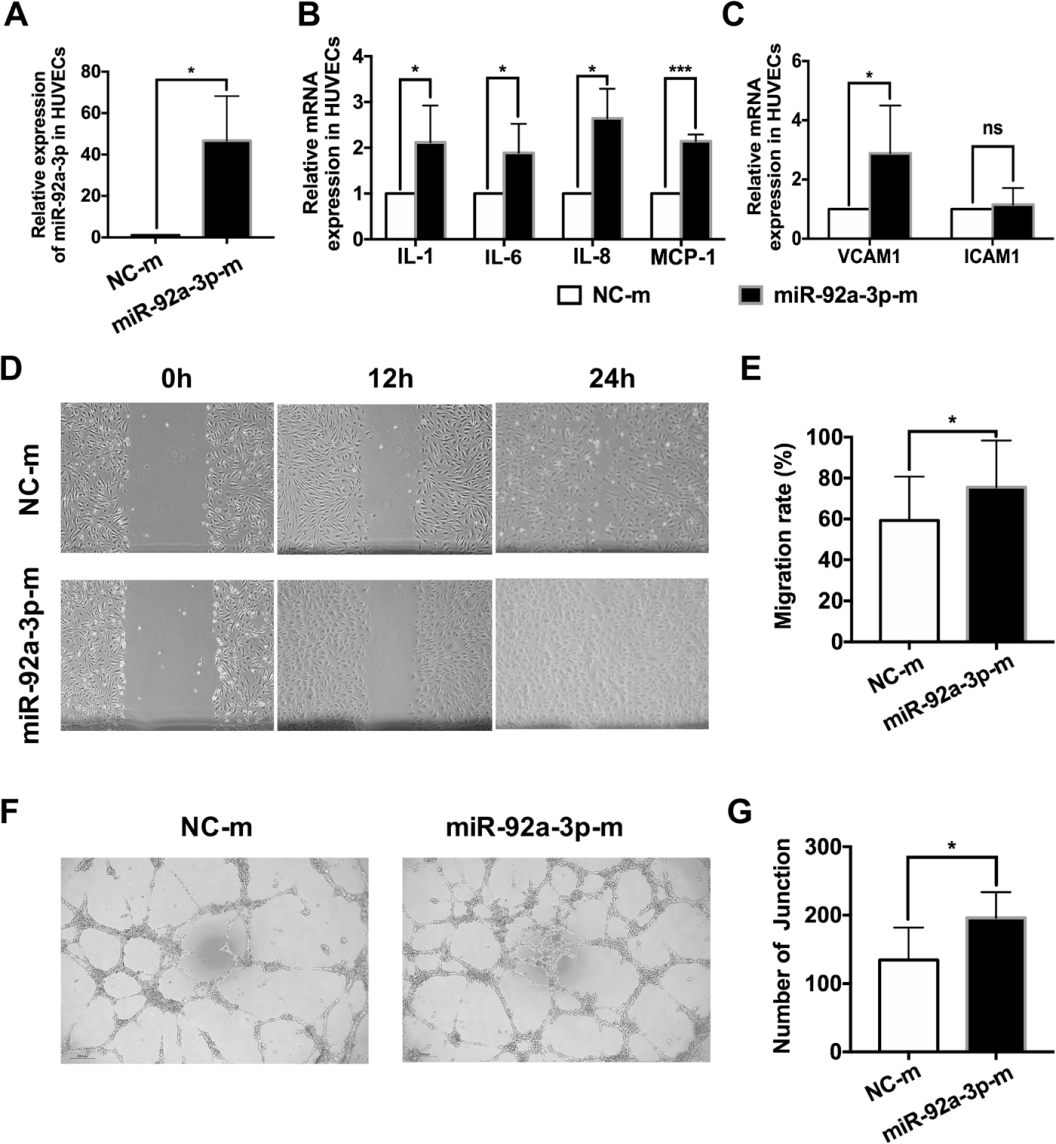
Overexpression of miR-92a-3p in HUVECs enhances angiogenesis in vitro. **A**, **B** HUVECs were transfected with synthetic mimic control (NC-m) or miR-92a-3p mimic (miR-92a-3p-m). The mRNA levels of miR-92a-3p, VCAM1, ICAM1, IL-1, IL-6, IL-8, and MCP-1 were increased (*n* ≥ 4, ^*^P < 0.05, ^**^P < 0.01, ^***^P < 0.001). **C** Wound-healing assay revealed that miR-92a-3p could enhance the migration rate of the HUVECs. **D** Relative quantification of HUVEC migration 24 h after the wound scratch (*n* = 4, ^*^P < 0.05). **E**, **F** Quantitative evaluation of the number of junctions after transfection of the HUVECs with NC-m or miR-92a-3p (24 h, *n* = 9, ^*^P < 0.05).

To further confirm the function of miR-92a-3p in angiogenesis, we directly transfected HUVECs with normal control mimics (NC-m) or miR-92a-3p mimics (miR-92a-3p-m). As shown in Fig. 6A, the miR-92a-3p level was significantly upregulated in the HUVECs transfected with miR-92a-3p (relative fold change: 46.68 ± 21.55-fold, *P < 0.05). The expression of inflammatory cytokines related to angiogenesis was also significantly increased in the HUVECs treated with miR-92a-3p-m compared to the HUVECs treated with the control (relative fold change: IL-1, 2.12 ± 0.81-fold; IL-6, 1.89 ± 0.63-fold; IL-8, 2.65 ± 0.65-fold; MCP-1, 2.15 ± 0.15-fold; *P < 0.05, ***P < 0.001) (Fig. 6B). Consistent with the results described above, miR-92a-3p-m only increased VCAM1 mRNA, not ICAM1 mRNA, in the HUVECs (relative fold change: 2.88 ± 1.62-fold. *P < 0.05) (Fig. 6C). However, miR-92a-3p-m also promoted the migration and tube formation of HUVECs compared to the control (24 h migration rate: NC-m, 59.32 ± 21.38%; miR-92a-3p-m, 75.51 ± 22.90%, *P < 0.05; number of junctions: NC-m, 134.44 ± 47.33; miR-92a-3p-m, 196.11 ± 37.50. *P < 0.05) (Fig. 6D–G). Therefore, these results support that miR-92a-3p could directly contribute to angiogenesis. Moreover, this evidence further confirms that miR-92a-3p in exosomes derived from RB cells might be involved in RB angiogenesis.

### Exosomally delivered miR-92a-3p modulates angiogenesis by targeting KLF2

**Fig. 7:**
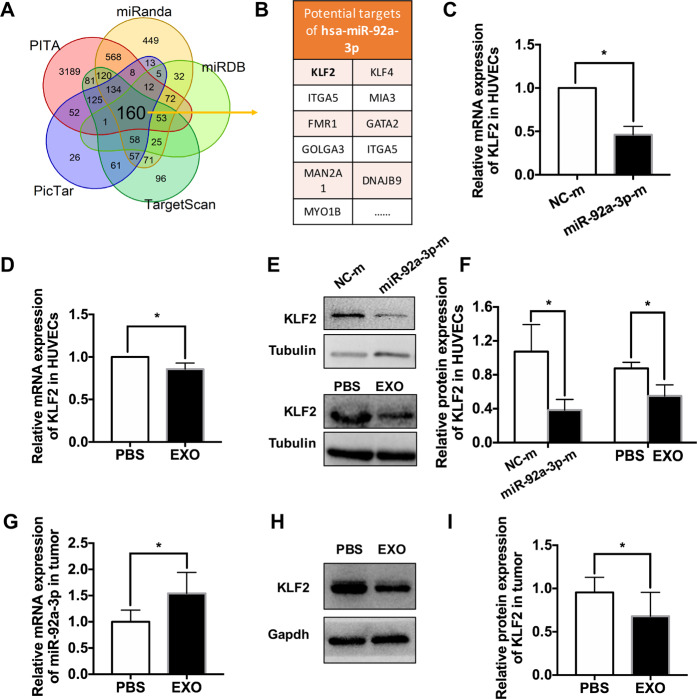
Exosomal delivered miR-92a-3p modulates angiogenesis by targeting KLF2. **A** Venn diagram showing overlap of the data from miRanda, miRDB, TargetScan, PicTar, and PITA. **B** Potential targets of hsa-miR-92a-3p. **C** The mRNA level of KLF2 in the HUVECs transfected with synthetic mimic control (NC-m) or miR-92a-3p mimic (miR-92a-3p-m) (*n* = 4, *P < 0.05). **D** The mRNA level of KLF2 in the HUVECs treated with PBS or EXOs (*n* = 5, *P < 0.05). **E** Western blot analysis and relative quantification of protein expression indicating that exosome treatment or overexpression of miR-92a-3p could downregulate KLF2 expression in HUVECs (*n* ≥ 6, *P < 0.05). **G** The mRNA level of miR-92a-3p in the tumor tissues treated with PBS or exosomes (*n* = 6, ^*^P < 0.05). **H** Representative blots of KLF2 in the tumor tissues treated with PBS or exosomes. **I** Quantitative evaluation of KLF2 protein expression in the tumor tissues treated with PBS or exosomes (*n* = 6, ^*^P < 0.05).

To further reveal the potential molecular mechanism underlying the proangiogenic activity of miR-92a-3p, we predicted the downstream target of miR-92a-3p by miRanda, miRDB, TargetScan, PicTar, and PITA. Based on the prediction results of the Venn diagram, we found that 160 genes might be related to miR-92a-3p (Fig. 7A). After screening as in previous studies, five genes (KLF2, KLF4, ITGA5, GATA2, and MIA3) were associated with the angiogenesis pathway. After RT-PCR analysis of these five genes (data were not shown), we focused on the Krüppel-like factor 2 (KLF2) (Fig. 7B). KLF2 has been proven to be the target of miR-92a-3p and plays an important role in tumor angiogenesis [15, 20]. Consistent with the prediction, the overexpression of miR-92a-3p significantly repressed the expression of KLF2 in HUVECs compared to the control (relative fold change: 0.46 ± 0.10-fold. *P < 0.05) (Fig. 7C). Moreover, KLF2 was also decreased in the HUVECs treated with the RB exosomes compared to PBS (relative fold change: 0.86 ± 0.07-fold. *P < 0.05) (Fig. 7D). Similarly, the western blotting results also showed that the protein expression of KLF2 was significantly decreased in the HUVECs in the miR-92a-3p-m group and exosome treatments group compared to those in the control groups (Fig. 7E). The results are presented in histograms (NC-m: 1.072 ± 0.320; miR-92a-3p-m, 0.383 ± 0.127, *P < 0.05. PBS, 0.875 ± 0.072; EXO, 0.550 ± 0.131, *P < 0.05) (Fig. 7F). Furthermore, the relative expression of miR-92a-3p and KLF2 was also confirmed in tumor tissues treated with PBS or exosomes. miR-92a-3p mRNA expression was increased, whereas KLF2 protein expression was decreased, in the exosome groups compared with the control group (relative fold change, miR-92a-3p: PBS, 1.00 ± 0.22, EXO, 1.54 ± 0.40; KLF2: PBS, 0.96 ± 0.17, EXO, 0.68 ± 0.28, *P < 0.05) (Fig. 7G–I).

**Fig. 8:**
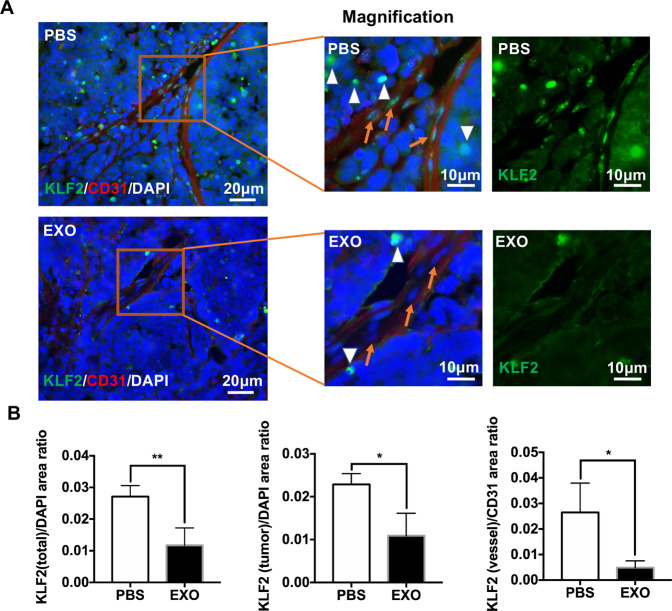
Exosomes derived from WERI-Rb1 cells decrease the expression of KLF2 both in tumor tissues and vessels. **A** CD31 (red) and KLF2 (green) were labeled by immunofluorescence in tumors from the PBS‐injected or EXO‐injection groups. White arrowheads: KLF2 in the tumor tissue, orange arrows: KLF2 in the vessels, scale bar, 20 or 10 μm. **B** Quantification data showed KLF2(total)/DAPI area ratio, KLF2(tumor)/DAPI area ratio, and KLF2(vessel)/CD31 area ratio from the PBS‐injected and EXO‐injected groups (*n* = 5, ^*^P < 0.05, ^**^P < 0.01).

In addition, double immunofluorescent staining with KLF2 (green) and CD31 (red) showed that injection of exosomes obviously resulted in downregulation of KLF2 in the tumor tissue (white arrowheads) and vessels (orange arrows), compared with the PBS group (Fig. 8A). Statistical data were presented in histograms (KLF2(total)/DAPI area ratio: PBS, 0.027 ± 0.0034; EXO, 0.012 ± 0.0055; KLF2(tumor)/DAPI area ratio: PBS, 0.023 ± 0.0025, EXO, 0.011 ± 0.0052; KLF2(vessel)/CD31 area ratio: PBS, 0.026 ± 0.012, EXO, 0.0049 ± 0.0026, *P < 0.05, **P < 0.01) (Fig. 8B). Thus, miR-92a-3p/KLF2 might be involved in exosome-mediated angiogenesis during RB progression.

## Discussion

In the present study, we investigated the effect of RB exosomes on angiogenesis both in vitro and in vivo. The evidence presented here indicates that exosomes derived from the RB WERI-Rb1 cell line could increase the cell viability, tube formation, and migration of HUVECs and induce an inflammatory response by increasing the expression of a series of related genes. Moreover, experiments with a nude mouse xenotransplantation model confirmed that exosomes significantly affected tumor development and angiogenesis. In addition, we showed that exosomes could upregulate miR-92a-3p expression in HUVECs and modulate angiogenesis by inhibiting KLF2. Thus, our study provided biochemical and functional evidence to indicate the angiogenic role of exosomes in RB progression.

First, our data showed that exosomes derived from WERI-Rb1 cells could be engulfed by HUVECs, accumulate around the nucleus, and induce an increase in the expression of inflammatory molecules (IL-1, IL-6, IL-8, and MCP-1). These results are consistent with previous reports. For example, IL-1 is required for angiogenesis and invasiveness in various tumors and serves as a vital molecule in the tumor microenvironment [21]. Maji et al. demonstrated that exosomes could activate IL-6 in breast cancer pathogenesis [22]. Elevated levels of IL-6, IL-8, and MCP-1 in lung cancer-derived exosomes significantly enhanced tumor growth [23]. Similarly, our previous study also indicated that RB exosomes could increase the expression of IL-6 and MCP-1 in both murine RAW264.7 macrophages and bone marrow mesenchymal stem cells, which inhibited their antitumor activity [13]. In addition, the expression of the cell adhesion molecules VCAM1 and ICAM1 was upregulated in HUVECs. These adhesion molecules on endothelial cells could stabilize cell–cell contact and were associated with the processes of tumor neovascularization and progression [24]. The results were confirmed in vivo (Figs. 3 and 4). Thus, these findings suggest that exosomes serve as an important mediator and influence different types of cells in the tumor environment.

In addition, angiogenesis of solid tumors plays an important role in their development by maintaining the oxygen and nutrient supply. A growing body of evidence suggests that exosome could promote tumor angiogenesis. For example, exosomes derived from HCC could enhance tumor angiogenesis [14]. Zeng et al. determined that cancer-derived exosomes could induce vascular permeability and angiogenesis to promote premetastatic niche formation [15]. Similarly, our data indicated that a much stronger blood flow signal, and more endothelial cells and blood vessels were observed in the xenotransplanted tumors treated with exosomes (Fig. 4). In addition to the proliferation and migration of endothelial cells, the process of angiogenesis also requires the recruitment of bone marrow-derived progenitor cells (BMDPCs) to the site of neovascularization [25]. Although we did not trace BMDPCs in tumor tissues at 3 weeks (data were not shown), there should be a strong association between exosomes and the recruitment of BMDPCs. The recruitment of BMDPCs induced by exosomes should occur at the early time points of tumor growth.

More recently, multiple miRNAs have been reported to be selectively packaged into exosomes and transferred to different target cells to modulate various physiological and pathological processes [26, 27]. In the present study, functional validation of the role of exosomal miR-92a-3p in HUVEC angiogenesis was verified by miR-92a-3p knockdown in exosomes and overexpression in HUVECs (Figs. 5 and 6). miR-92a-3p, belongs to the miR 17~92 cluster and has been reported to facilitate colon cancer angiogenesis through the inhibition of Dickkopf-3 [28]. In addition, exosomal miR-92a-3p promoted partial endothelial-mesenchymal transition, which was associated with the induction of cancer development [29]. In particular, miR-92a-3p has been reported to be highly expressed in RB and to accelerate its progression [30]. Therefore, this evidence indicates that miR-92a-3p plays crucial roles in the proangiogenic activity and progression of RB through exosomes. Nevertheless, ICAM1 expression showed no significant difference when miR-92a-3p was overexpressed in HUVECs, whereas it was upregulated in HUVECs treated with exosomes treatment and downregulated in HUVECs treated with exosomes transfected with the miR-92a-3p inhibitor. We speculate that there are other molecules within the RB exosomes, in addition to miR-92a-3p, could influence ICAM1 expression.

Finally, bioinformatics analysis predicted that KLF2 could be a potential target of miR-92-3p. The mRNA and protein levels of KLF2 were decreased in HUVECs treated with exosomes or the miR-92a-3p mimic (Fig. 7). KLF2 protein level was also decreased in tumor tissues. Thus, miR-92a-3p/KLF2 might be involved in the angiogenesis of RB. This notion is also supported by previous studies. KLF2 belongs to the KLF family, a subclass family of zinc-finger-containing transcription factors characterized by a DNA-binding domain, and modulates tumor proliferation, apoptosis, metastasis, and microenvironment [31]. KLF2 could impede angiogenesis by inhibiting the promoter activity of VEGFR2 [32], whereas the downregulation of KLF2 by exosomal miR-25-3p could promote vascular permeability and angiogenesis in CRC [15].

In summary, this study provided direct evidence supporting the notion that exosomes derived from RB play an important role in tumor angiogenesis and that exosomal miR-92a-3p might be a novel therapeutic target during RB deterioration.

## Materials and methods

### Cell culture

The human RB cell line, WERI-Rb1, was purchased from the American Type Culture Collection (ATCC, Manassas, VA, USA) and cultured in RPMI-1640 (Gibco, CA, USA) with 10% fetal bovine serum (FBS). HUVECs (ScienCell, CA, USA) were cultured in ECM (ScienCell, CA, USA) with 1% ECGS and 5% FBS. All the cells were incubated in a humidified atmosphere with a mixture of 1% O_2_, 5% CO_2_, and 94% N_2_ at 37 °C.

### Exosome purification

Exosomes were isolated from WERI-Rb1-derived conditioned medium after 2 days of culture via sequential differential centrifugation [33]. Briefly, conditioned media were centrifuged at 1000 × *g* for 5 min and 10,000 × *g* for 30 min at 4 °C. Then, the supernatants were ultracentrifuged at 100,000 × *g* for 90 min at 4 °C. Finally, the exosomes were washed with phosphate-buffered saline (PBS) followed by another ultracentrifugation at 100,000 × *g* for 90 min at 4 °C. In addition, exosomes were resuspended in PBS and the protein concentration was determined by a bicinchoninic acid Protein Assay Kit (BCA, Beyotime, Jiangsu, China). For cell treatment, 1 × 10^4^ recipient cells were incubated with 10 μg of exosomes.

### Characterization of exosomes

For transmission electron microscopy, as outlined previously [33], freshly collected exosomes were infiltrated with a copper grid coated with carbon in a drop of 1% glutaraldehyde for 5 min. The samples were washed three times in distilled water and were then stained with 4% uranyl‐acetate solution for 10 min at 37 °C. The samples were then treated with a drop of methyl cellulose for 5 min on ice and washed with distilled water before observation under an FEI Tecnai Spirit G2 transmission electron microscope (Thermo Fisher Scientific, Inc.), which was operated at 80 kV.

For NTA, NTA is a method used for the detection of secreted EVs in a liquid sample. Exosomes were analyzed using ZetaView Particle Metrix (ZetaView PMX 110). The samples were diluted at 1:100 in PBS and analyzed.

### Cell viability assay

The viability of HUVECs treated with exosomes or PBS was detected by a Cell Counting Kit-8 (CCK-8) assay (Dojindo, Japan) according to the manufacturer’s protocol. Subsequently, the absorbance at 450 nm was assessed, and the absorbance was recorded after incubation with CCK-8 reagent for 3 h at 37 °C.

### Real-time PCR assay

Total RNA was extracted from of HUVECs, WERI-Rb1 cells and tumor tissues by TRIzol Reagent (Invitrogen, Carlsbad, CA) and subjected to reverse transcription using a PrimeScriptTM RT Reagent kit (Takara Biotechnology Co., Ltd), according to the manufacturer’s protocol. For exosome RNA extraction, TRIzol^®^ reagent (Invitrogen; Thermo Fisher Scientific, Inc.) and Dr GenTLETM Precipitation Carrier (Takara Biotechnology Co., Ltd) were used, and a Mir-XTM miRNA First-Strand Synthesis Kit (Takara Biotechnology Co., Ltd) was used for reverse transcription. The mRNA expression was detected by real-time PCR using a SYBR Prime Script RT-PCR Kit on a Roche 480 system (Roche Diagnostics) following the manufacturer’s protocols. The PCR conditions were as follows: 94 °C for 5 min, followed by 40 cycles of 94 °C for 30 s, 62 °C for 30 s, and 72 °C for 30 s. The relative target gene expression was calculated using the ΔΔCt method [34] and normalized to the endogenous expression of b-actin, GAPDH or miR-16. The following primer pairs were used: VCAM1, 5′-CGAAAGGCCCAGTTGAAGGA-3′ (sense) and 5′-GAGCACGAGAAGCTCAGGAGAAA-3′ (antisense); ICAM1, 5′-AGCCAACCAATGTGCTATTCAAAC-3′ (sense) and 5′-CACCTGGCAGCGTAGGGTAA -3′ (antisense); MCP-1, 5′-CAGCCAGATGCAATCAATGCC-3′ (sense) and 5′-TGGAATCCTGAACCCACTTCT-3′ (antisense); IL-1, 5′-ATGATGGCTTATTACAGTGGCAA -3′ (sense) and 5′- GTCGGAGATTCGTAGCTGGA -3′ (antisense); IL-6, 5′- ACTCACCTCTTCAGAACGAATTG-3′ (sense) and 5′-CCATCTTTGGAAGGTTCAGGTTG-3′ (antisense); IL-8, 5′-GCATAAAGACATACTCCAAACC-3′ (sense) and 5′-ACTTCTCCACAACCCTCTG-3′ (antisense); KLF2, 5′-CTACACCAAGAGTTCGCATCTG-3′ (sense) and 5′-CCGTGTGCTTTCGGTAGTG-3′ (antisense);GAPDH, 5′-GAGTCAACGGATTTGGTCGT-3′ (sense) and 5′-CATGGGTGGAATCATATTGGA-3′ (antisense). ACTIN, 5′-CACCACACCTTCTACAATGAG-3′ (sense) and 5′-TAGCACAGCCTGGATAGCAAC-3′ (antisense). microRNA-92a-3p, 5′-UAUUGCACUUGUCCCGGCCUGU -3′ (sense).

### Immunofluorescence assay

Cells or tissue sections were fixed with ice-cold 4% paraformaldehyde for 10 min at 37 °C and then blocked with 1% bovine serum albumin and 0.2% Triton X-100 for 30 min. The samples were first incubated with primary antibodies, including anti-CD31 (1:250; cat. no. ab28364, ab24590 Abcam, Cambridge, MA, USA), anti-KLF2 (1:100; cat. no. a16480 ABclonal Technology, MA, USA) and anti-isolectin B4 (1:500; cat. no. I121411; B4-594, Molecular Probes, Carlsbad, CA, USA). Then, the samples were stained with secondary antibodies for 1 h at 37 °C (Alexa Fluor 555 anti-rabbit IgG, 1:500, cat. no. 4413, Cell Signaling Technology, Inc.).

### Western blotting

Cells, exosomes and tissues were lysed in RIPA buffer (Biotech, Beijing, China). The protein concentration was measured via the BCA Protein Assay Kit (Beyotime, Jiangsu, China). Western blotting was performed with standard protocols. Primary antibodies against the following antigens were used: anti-GAPDH (1:10,000; cat. no. 10494-1-AP; ProteinTech Group, Inc.), anti-Tubulin (1:1000; cat. no. sc-5274; Santa Cruz Biotechnology, Inc.), anti-CD63 (1:1000; cat. no. EXOAB-CD63A-1; Systems Biosciences, LLC), antitumor susceptibility gene 101 protein (1:1000; cat. no. ab125011; Abcam), anti-CD9 (1:1000; cat. no. 13403; Cell Signaling Technology, Inc.), anti-heat shock 70 kDa protein 1A (1:1000; cat. no. 4873; Cell Signaling Technology, Inc.), anti-VCAM1 (1:1000; cat. no. SC-13160; Santa Cruz, CA, USA), anti-ICAM1 (1:1000; cat. no. SC-8439; Santa Cruz, CA, USA), anti-CD31 (1:1000; cat. no. ab28364 Abcam, Cambridge, MA, USA), and anti-KLF2 (1:1000; cat. no. PA5-40591 Invitrogen, Thermo Fisher Scientific, Inc.). Following incubation with HRP-conjugated anti-mouse IgG (1:3,000; cat. no. 7076s; Cell Signaling Technology, Inc.) or anti-rabbit IgG (1:10000; cat. no. 7074s; Cell Signaling Technology, Inc.) for 1 h at 37 °C, and the membranes were visualized with an enhanced chemiluminescence system (Thermo Fisher Scientific, Inc.) according to the manufacturer’s protocol.

### Wound healing and tube formation assay

A total of 4 × 10^4^ HUVECs were seeded in a 12-well plate and treated with PBS or different exosomes for 24 h. Then, the cell monolayer was scraped with a P200 pipet tip. Images of the wound spaces were captured at 0, 12, and 24 h and analyzed by Image-Pro Plus software. For tube formation, Matrigel (Corning) was used to coat 96-well plates and incubated at 37 °C for 30 min to allow the Matrigel to polymerize. After treatment with PBS or different exosomes, a total of 4 × 104 HUVECs were seeded in the Matrigel-coated wells. The plates were then incubated at 37 °C in a 5% CO_2_ humidified atmosphere. After 12 h, tube formation was visualized with a microscope and determined by measuring the number of tubes. Each experiment was repeated three times.

### Murine xenograft model of retinoblastoma

Twenty 4–6-week-old female athymic nude mice were subcutaneously injected in the left submaxillary region with 300 μl of 5 × 10^6^ WERI-Rb1 cells mixed with Matrigel (vol/vol, 1:1). After 1 week, successfully transplanted mice were injected with an equal volume of PBS or exosomes (50 μg) into the primary tumor site every 3 days for 21 days. The tumor sizes of the mice were measured, and the tumor volumes were calculated every 5 days with the following formula: length × width^2^/2. The tumors were harvested 21 days after injection and stored at −80 °C.

### Ultrasound analysis

Ultrasound analysis of the blood flow in tumors was detected by a small-animal high-resolution imaging unit (Vevo 770; VisualSonics) before the mice were sacrificed. The tumors were measured both on the short and long axes, and the ratio of blood flow in each ultrasound image was quantified by ImageJ software.

### miRNA-targets prediction

miRNAs obtained from previous experimental results were selected and analyzed with miRWalk2.0 to predict their targets. For the purpose of improving the accuracy of prediction results, five popular databases, TargetScan, miRanda, miRDB, PITA, and PicTar, were used to perform intersection. The target genes identified by all the databases were selected for further analysis.

### miRNA mimics and miRNA inhibitor transfection

HUVECs at 70–80% confluency were transfected with 75 pM miRNAs using Lipofectamine 3000 in Opti-MEM (Invitrogen) according to the manufacturer’s procedures. The synthetic mimic control (NC-m) and miR-92a-3p mimic (miR-92a-3p-m) were purchased from RiboBio (RiboBio Co. Ltd, Guangzhou, Guangdong). After transfection for 12 h, the culture medium was replaced with ECM (ScienCell, CA, USA) with 1% ECGS and 5% FBS. Exosomes were transfected with synthetic inhibitor control (NC-i) or miR-92a-3p inhibitor (miR-92a-i) via calcium chloride transfection according to a previous study [35].

### Statistical analysis

The data are represented as the means ± standard deviation, and the differences between mean values were evaluated using Student’s two-tailed *t* test (for two groups). The data were considered significantly different at a *P* value < 0.05.
